# A promising strategy for combating bacterial infections through the use of light-triggered ROS in Ce6-immobilized hydrogels

**DOI:** 10.1093/rb/rbae101

**Published:** 2024-08-23

**Authors:** Seung Hee Hong, Mi Hee Lee, Eun Jeong Go, Jong-Chul Park

**Affiliations:** Department of Medical Engineering, Yonsei University, College of Medicine, Seoul 03722, Republic of Korea; Graduate School of Medical Science, Brain Korea 21 Project, Yonsei University, College of Medicine, Seoul 03722, Republic of Korea; Department of Medical Engineering, Yonsei University, College of Medicine, Seoul 03722, Republic of Korea; Department of Medical Engineering, Yonsei University, College of Medicine, Seoul 03722, Republic of Korea; Department of Medical Engineering, Yonsei University, College of Medicine, Seoul 03722, Republic of Korea; Graduate School of Medical Science, Brain Korea 21 Project, Yonsei University, College of Medicine, Seoul 03722, Republic of Korea

**Keywords:** hyaluronic acid hydrogel, ROS, antibacterial effect, macrophage polarization

## Abstract

The reactive oxygen species (ROS) are composed of highly reactive molecules, including superoxide anions ($O_{2}^{•-}$), hydrogen peroxide (H_2_O_2_) and hydroxyl radicals. Researchers have explored the potential benefits of using hydrogel dressings that incorporate active substances to accelerate wound healing. The present investigation involved the development of a hyaluronic acid (HA) hydrogel capable of producing ROS using LED irradiation. The process of creating a composite hydrogel was created by chemically bonding Ce6 to an amide group. Our analysis revealed that the synthesized hydrogel had a well-structured amide bond, and the degree of cross-linking was assessed through swelling, enzyme stability and cytotoxicity tests. ROS production was found to be influenced by both the intensity and duration of light exposure. Furthermore, in situations where cell toxicity resulting from ROS generation in the hydrogel surpassed 70%, no detectable genotoxic consequences were evident, and antibacterial activity was confirmed to be directly caused by the destruction of bacterial membranes as a result of ROS damage. Furthermore, the utilization of the generated ROS influences the polarization of macrophages, resulting in the secretion of pro-inflammatory cytokines, which is a characteristic feature of M1 polarization. Subsequently, we validated the efficacy of a HA hydrogel that produces ROS to directly eradicate microorganisms. Furthermore, this hydrogel facilitated indirect antibacterial activity by stimulating macrophages to release pro-inflammatory cytokines. These cytokines are crucial for coordinating cell-mediated immune responses and for modulating the overall effectiveness of the immune system. Therefore, the Ce6-HA hydrogel has the potential to serve as an effective wound dressing solution for infected wounds because of its ability to produce substantial levels or a consistent supply.

## Graphical Abstract

**Figure:**
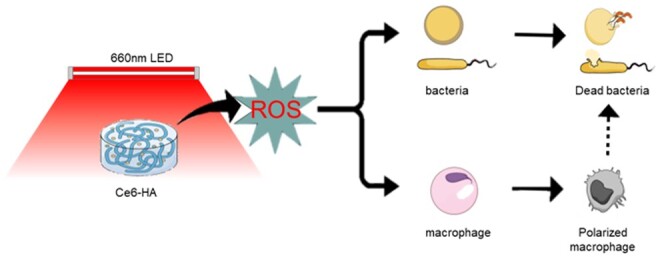

## Introduction

The skin is the body’s most extensive organ and functions as a protective barrier against external environmental factors [1–3]. When injury occurs, a complex healing process is initiated that involves hemostasis, inflammation, proliferation and tissue remodeling [4, 5]. The field of biomedical engineering is constantly evolving with a primary focus on the creation of new materials and technologies to address pressing healthcare concerns [6]. The field of wound healing is continually being explored to discover new materials that can facilitate healing and protect against infections. The selection of materials plays a crucial role in wound healing as it affects the rate of recovery, prevention of infections and comfort experienced by the patient [7, 8]. One significant aspect of the relevance of materials in the process of wound healing is their capacity to establish a conducive environment that facilitates the body’s inherent healing processes. Hydrogels are widely studied and applied because of their ability to maintain a moist environment at the wound site, which promotes faster healing [9]. Ensuring biocompatibility is of utmost importance because it guarantees that these materials do not elicit unfavorable reactions within the body. Hyaluronic acid (HA) is a naturally occurring polysaccharide found in connective tissues that typically does not form amide bonds in its unadulterated state. Instead, it is primarily composed of alternating D-glucuronic acid and *N*-acetyl-d-glucosamine units connected by β(1 → 4) and β(1 → 3) glycosidic bonds, resulting in a linear structure [10, 11].

Hydrogel dressings are vital for facilitating effective wound healing because of their ability to create a moist environment that supports tissue regeneration. These dressings, composed of cross-linked HA molecules, have a remarkable capacity for water absorption and possess biocompatibility and non-immunogenicity, making them suitable for treating a wide range of wounds, including chronic wounds and burns [12, 13].

Chemical modifications can facilitate the formation of amide bonds with HA molecules, enabling the attachment of various functional groups, peptides or pharmaceuticals. This broadens their potential use in drug delivery, tissue engineering and other biomedical applications [14]. The procedure involves activating HA’s carboxyl groups of HA using agents such as carbodiimides or *N*-hydroxysuccinimide to react with primary amines and form stable amide bonds. This amidation modifies the physical and chemical properties and biological functions of HA, transforming it into an adaptable biomaterial with customized characteristics for specific purposes [15]. In this procedure, HA molecules undergo modifications that incorporate functional groups capable of forming amide bonds with specific reactive sites on a photodynamic agent. Consequently, a long-lasting covalent connection was established between the photodynamic agent and the HA backbone. The formation of amide bonds leads to the creation of a composite material that preserves the natural properties of HA, such as biocompatibility and biodegradability, while incorporating the photo-responsive features of the photodynamic agent.

Incorporation of photosensitizers into HA-based hydrogels offers several benefits. The hydrogel matrix functions as a support for the controlled release of photosensitizers, ensuring their targeted and sustained delivery to specific sites [15]. This allows for the precise release of ROS when exposed to light, enabling focused applications in wound healing and tissue regeneration through photodynamic therapy (PDT) [16, 17]. PDT demonstrates significant potential for addressing various conditions, such as cancer and infectious diseases. When activated by specific wavelengths of light, photosensitizers can produce ROS that effectively eliminate target cells or pathogens [18]. PDT involves administering a photosensitizer, such as porphyrin derivatives or chlorin e6, followed by exposure to light of an appropriate wavelength to generate ROS [19]. Upon activation by light, the photosensitizer absorbs photons and undergoes a sequence of photochemical reactions, leading to the production of singlet oxygen (^1^O_2_), which exhibits potent antibacterial effects. ROS-based antibacterial approaches, such as PDT, show significant potential for addressing multidrug-resistant pathogens and overcoming obstacles related to traditional antibacterial treatment. The use of ROS for bacterial eradication presents an innovative avenue for managing infectious diseases, wound infections and biofilm-related infections across different medical environments [20, 21].

**Figure 1.**
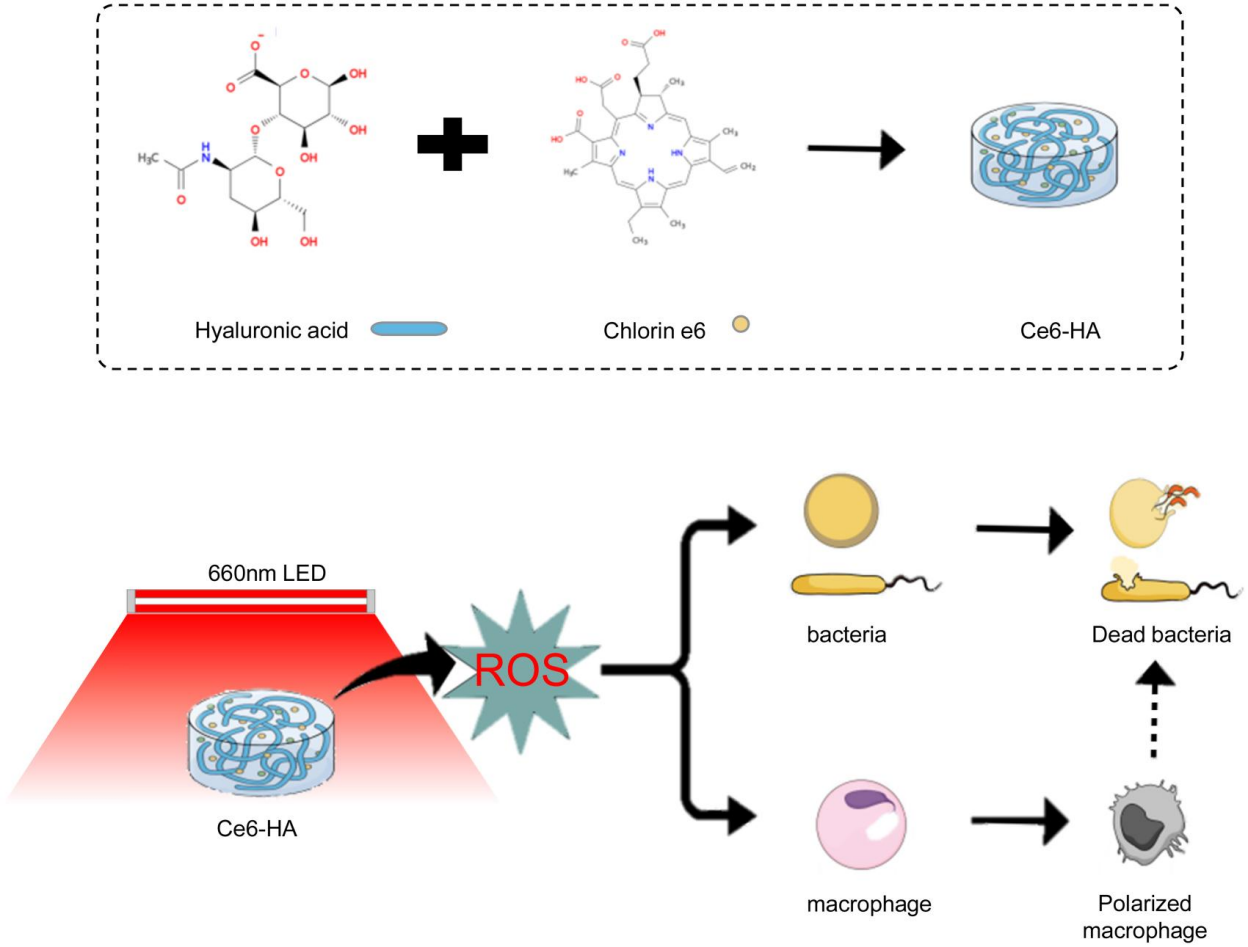
Schematic of the activities promoted by the Ce6-HA antibacterial effect.

The goal of this research is to develop a sophisticated hydrogel system that incorporates photosensitizer molecules and HA, in order to produce ROS when exposed to light. The primary objective of this study is to comprehensively analyze the impact of this novel approach on enhancing the antibacterial efficacy against a variety of pathogenic bacteria (Figure 1).

## Materials and methods

### Preparation of Ce6-HA hydrogel

**Figure 2.**
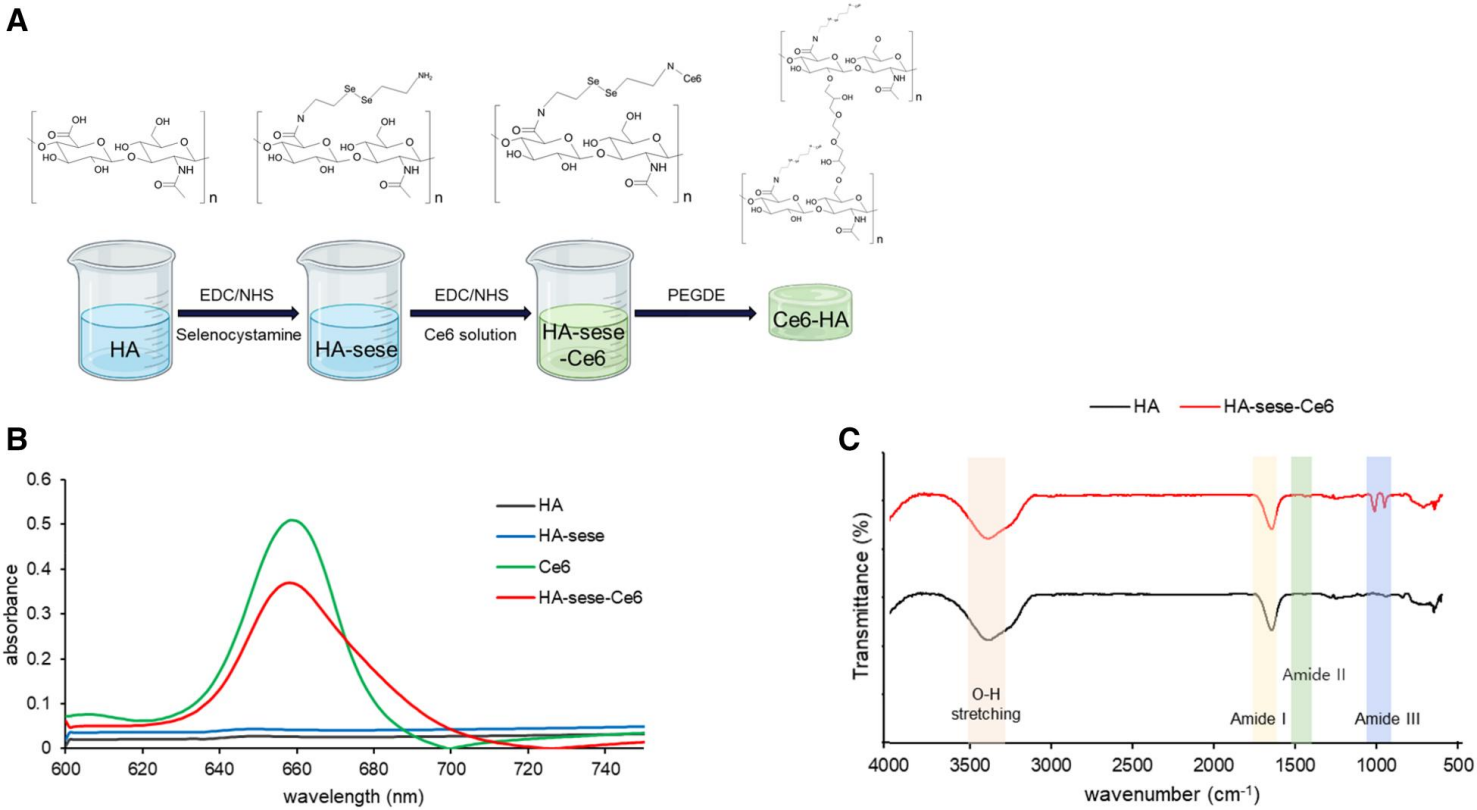
Fabrication and characterization of HA-sese-Ce6. (**A**) Schematic of the Ce6-HA fabrication method. (**B**) UV-Vis spectra of HA, HA-sese, HA-sese-Ce6 and Ce6 solutions from 600 to 750 nm. (**C**) FTIR spectra of the Ce6-HA-bonded hydrogel formed by EDC/NHS coupling. From top to bottom: HA-sese-Ce6 and HA spectra shown as % transmittance.

Sodium HA was purchased from Lifecore Biomedical (USA, 200 kDa), while EDC, NHS, selenocystamine dihydrochloride (C_4_H_12_N_2_Se_2_·2HCl) and polyethylene glycol diglycidyl ether (PEGDE) were acquired from Sigma-Aldrich (St Louis, MO, USA). Chlorin e6 (Ce6) was procured from Santa Cruz Biotechnology (USA), and Dulbecco’s phosphate-buffered saline (DPBS) was purchased from Biosesang (Korea). Ce6-HA was synthesized using carbodiimide coupling chemistry. Briefly, HA was dissolved in PBS buffer. Next, EDC, NHS and selenocystamine were gradually added to the dissolved HA solution and stirred at room temperature for 4–6 h. Ce6 was dissolved in DMSO at 5 mg/ml, after which 0.9 ml of a solution of Ce6 was introduced into the mixture and left to mix overnight. The mixed solution was then transferred to an ultrafiltration centrifuge tube (Pall Corporation, USA) and the sample was centrifuged at 4500 rpm at 20°C for 20 min. The ultrafiltration centrifuge tube was rinsed three times with deionized water to eliminate any uncombined HA and Ce6. After washing, the crosslinker PEGDE was introduced and the blend was transferred to a petri dish, which was then dried at room temperature. The Ce6-HA hydrogel formed a 1 cm^2^ circle, which was sterilized using 70% ethanol and then rinsed with distilled water (DW) (Figure 2A).

### Characterization of Ce6-sese-HA

#### UV-vis spectrum

UV spectroscopy was employed to verify the presence of Ce6 in the synthesized compound. Samples from each stage of the synthesis were evaluated. The optical densities of the HA, HA-sese, HA-sese-Ce6 and Ce6 solutions were examined within the wavelength range of 600–750 nm using a UV-Vis spectrophotometer.

#### FTIR

FTIR (Vertex70, Bruker, MA, USA) equipped with an attenuated total reflectance accessory was used to examine the chemical compositions of the HA and HA-sese-Ce6 solutions. Spectral data ranging from 4000 to 500 cm^−1^ were obtained by averaging 100 scans at a resolution of 4 cm^−1^.

### Crosslinker concentration determination

#### Swelling ratio

Different molar ratios of PEGDE were utilized: 0.3 mol PEGDE/1 mol HA, 0.5 mol PEGDE/1 mol HA and 0.7 mol PEGDE/1 mol HA. The swelling ratio was assessed to determine the appropriate amount of the crosslinking agent required. The produced HA and Ce6-HA hydrogels with various molar ratios of the crosslinker were weighed using an analytical balance and then immersed separately in PBS for 48 h at room temperature. Subsequently, the final weights were measured to calculate the swelling ratios by comparing their weights before and after immersion in PBS.

#### Enzyme stability

To assess the rate of deterioration of hydrogels exposed to hyaluronidase, a solution containing 100 units of hyaluronidase derived from bovine testes was prepared by dissolving it in PBS. The initial weights of the HA and Ce6-HA hydrogels were recorded before gently introducing hyaluronidase enzyme solution to the gels. Subsequently, their weights were measured at intervals of 0.5, 1, 2, 8, 12, 24, 48 and 72 h to determine the degradation profiles by calculating the remaining weights as percentages.

#### Crosslinking agent cytotoxicity

To evaluate the potential toxicity of Ce6-HA, NHDF at a density of 5 × 10^4^/cm^2^ and HaCaT cells at a density of 1 × 10^5^/cm^2^ were seeded in a well plate. Following cell attachment, cross-linked forms of Ce6-HA (cross-linked with varying molar ratios of PEGDE) were introduced into the wells, followed by incubation at 37°C for 1 day. Cell viability was assessed using the MTT reagent, which was added to each well and incubated in the dark for 4 h. Subsequently, DMSO was added to dissolve the cells and release the purple formazan crystals. The resulting combinations were transferred to a 96-well plate and the absorbance at 570 nm was measured using a microplate reader.

### ROS generation confirmation through DPBF

The catalytic activity of Ce6-HA was evaluated using 1,3-diphenylisobenzofuran (DPBF; Sigma-Aldrich) as an indicator of reactive oxygen. The Ce6-HA samples were placed in a DPBF solution and exposed to LED light with intensities of 1, 5, 10 or 20 mW/cm^2^ at a wavelength of 660 nm for 5 min intervals.

### Toxicological assessment of ROS generated by Ce6-HA

#### Cytotoxicity

NHDF were initially seeded in a well plate at a density of 5 × 10^4^ cells/well and then incubated overnight at 37°C with controlled humidity in a CO_2_ incubator. Subsequently, Ce6-HA was added to each well and the cells were exposed to red LED light with varying intensities ranging from 5 to 30 mW/cm^2^ for durations between 10 and 60 min. Cell viability after LED light exposure was assessed using the MTT assay. The MTT reagent was added to the wells and incubated for 4 h in a dark CO_2_ environment. Subsequently, DMSO was added to release the formazan crystals, and the absorbance was measured at 570 nm. The percentage of cell viability compared to that of the control group was used as an evaluation criterion.

#### Genotoxicity

To determine whether genetic mutations were induced by LED light, Ce6 hydrogel and ROS from Ce6-HA, we carried out the Ames test using *Salmonella typhimurium* strains TA98, TA100, TA1535, TA1537 and *Escherichia coli* WP2uvrA(pKM101). These bacterial strains were obtained from Moltox and cultured in a nutrient broth at 37°C. Each bacterial strain underwent specific treatment: HA combined with LED, Ce6-HA without LED and Ce6-HA with LED. The intensity of the LED was set to 10 mW/cm^2^ for 20 min. Mutagenicity was evaluated using a pre-incubation method. For each assay, negative controls containing saline and positive controls containing NQNO, sodium azide, ICR191 or MMS were prepared. Subsequently, histidine/biotin or tryptophan solutions were added to the treated bacterial suspensions and incubated in a shaking incubator at 37°C for 20 min. Subsequently, the top agar was mixed with these combinations before being transferred onto minimal agar plates. The resulting mixtures were then placed in an incubator at 37°C for 48 h, and the revertant colonies on each plate were manually counted. This process was repeated three times to validate the results.

### Antibacterial effect

#### Colony counting assay

To evaluate the antibacterial effects of HA and Ce6-HA when exposed to different levels of LED light, *Staphylococcus aureus* (ATCC 14458) and *Pseudomonas aeruginosa* (ATCC 9027) were employed in this study. Bacteria were cultured using the standard agar method at 37°C for 18 h. Bacterial suspensions at a concentration of 5 × 10^6^ CFU/ml in saline solution were prepared and treated with either HA or Ce6-HA. LED exposure comprised intensities of 5 mW/cm^2^ for durations ranging from 10 to 30 min, and 10 mW/cm^2^ for periods spanning from 10 to 20 min. Next, the bacterial suspensions were diluted with saline, spread onto plate count agar and allowed to dry before being incubated overnight at 37°C. Colony counts were determined after incubation.

#### Fluorescence assay

Assessment of the ratio of viable to non-viable *S.aureus* and *P.aeruginosa* after ROS treatment involved fluorescence staining. *S.aureus* and *P.aeruginosa* were treated with either HA or Ce6-HA, followed by LED light exposure at intensities ranging from 5 to 10 mW/cm^2^ for 10–30 min. Subsequently, the suspensions of *S.aureus* and *P.aeruginosa* were stained using the BacLight LIVE/DEAD kit at room temperature for 15 min. The resulting mixtures were transferred to black 96-well plates and scanned using a microplate reader. Excitation took place at a wavelength of 485 nm, with emissions recorded at 530 nm (green) or 630 nm (red).

#### Bacterial infection *ex vivo* model

The effectiveness of the antibacterial activity was evaluated by assessing the eradication of *S.aureus* and *P.aeruginosa* on porcine skin. The porcine skin was trimmed to a size of 1 cm^2^, and a silicone o-ring was used to minimize bacterial suspension loss. The bacterial suspension was applied to the prepared skin, followed by application of either HA or Ce6-HA. A red LED with power intensities ranging from 5 to 10 mW/cm^2^ was exposed for durations varying from 10 to 30 min. Subsequently, the bacterial suspension samples were collected, spread onto agar plates and incubated at 37°C overnight. Each experimental group was subjected to three replicates, and the colonies were quantified after incubation.

#### Malondialdehyde assay

The malondialdehyde (MDA) assay kit (Sigma Aldrich) was employed to assess oxidative damage in bacteria. *S.aureus* and *P.aeruginosa* suspensions were subjected to HA or Ce6-HA, followed by LED exposure at 5 mW/cm^2^ for 10–30 min, and 10 mW/cm^2^ for 10–20 min. The treated samples were lysed and centrifuged at 13 000 × g for 10 min, and the resulting supernatants were mixed with freshly prepared TBA solution to create MDA-TBA adducts. Subsequently, the samples were incubated at 95°C for 1 h before being cooled in an ice bath for 10 min to room temperature. The fluorescence signal strength of both the control and experimental samples was measured using a fluorescence microplate reader (Flexstation3, Molecular Devices) with an excitation wavelength of 532 nm and an emission wavelength of 553 nm.

#### Scanning electron microscopy

The bacterial shape and structure were examined using field-emission scanning electron microscopy (FE-SEM; S-800, Hitachi, Tokyo, Japan) at an acceleration emission voltage of 20 kV. *S.aureus* and *P.aeruginosa* suspensions, whether stimulated by ROS or not, were passed through filters with a pore size of 0.2 µm before being subjected to SEM imaging.

### ROS-induced macrophage polarization

#### Inflammatory cytokine analysis

RAW 264.7, cells were obtained from the American Type Culture Collection (ATCC^®^). ELISA kits (R&D Systems) were used to measure the levels of TNF-α, IL-1β, IL-6, IL-10 and TGF-β. The RAW 264.7 cells were cultured in a dish at a concentration of 1 × 10^5^ cells/ml until they reached full coverage. Subsequently, the hydrogel was applied to the cells, followed by irradiation with LED light at intensities ranging from 1 to 5 mW/cm^2^ for 10–20 min. To confirm the effects of low-power LED stimulation, an intensity of 100 μW/cm^2^ was applied for 30 and 60 min. Cell suspensions were centrifuged, and supernatants were collected to measure cytokine levels. The concentrations of pro-inflammatory cytokines (TNF-α, IL-1β, IL-6) and anti-inflammatory cytokines (TGF-β, IL-10) in the supernatants were assessed using ELISA kits according to the manufacturer’s guidelines and standard curves.

#### Protein level confirmation

RAW 264.7, cells were exposed to a hydrogel treated with LED power irradiation at intensities ranging from 1,2 and 5 mW/cm^2^ for varying durations between 10 and 20 min. ROS-activated cells were harvested and lysed using RIPA buffer on ice for 10 min, followed by collection with a scraper. The resulting mixture was centrifuged at 14 000 × g, at a temperature of 4°C for 15 min to collect the supernatant. The protein concentration was determined using a Bradford protein assay kit (Thermo Fisher Scientific). Subsequently, the samples were denatured at 95°C for 5 min. Equivalent quantities of protein samples were separated by sodium dodecyl sulfate-polyacrylamide gel electrophoresis (SDS-PAGE) before being transferred onto polyvinylidene fluoride (PVDF) membranes. The PVDF membrane was blocked with a 5% bovine serum albumin (BSA) solution for 1 h and then incubated with primary antibodies at 4°C overnight. Following washing, the membranes were incubated with secondary antibodies for 60 min at room temperature. The membranes were visualized using an ECL substrate (Cell Signaling Technology, Danvers, MA, USA). The primary antibodies used in this study were iNOS, CD86 and β-actin.

### Statistical analysis

Statistical analyses were conducted using SPSS 26.0 (SPSS Inc.). The data underwent one way analysis of variance (ANOVA) and also Student’s *t*-test for individual comparison within groups. Statistical significance was determined by *P* values below 0.05, and the data are presented as means ± standard deviations.

## Results and discussion

### Properties of Ce6-sese-HA

The UV-Vis spectra of HA, HA linked with selenocystamine, Ce6 solution and the resulting solution of HA-sese-Ce6 were used to validate the synthesis of HA connected with Ce6. The absorbance values were recorded in the range of 600–750 nm. A distinct absorbance peak for Ce6 was observed at 660 nm, whereas no absorbance peaks were detected for HA and HA-sese within the same range (Figure 2B). Notably, in the case of HA-sese-Ce6, an observable absorbance peak at 660 nm was present, confirming the successful conjugation of Ce6 with HA-sese [15, 22].

FTIR analysis was performed to confirm the covalent bonding between Ce6 and HA in HA-sese-Ce6 and to evaluate the binding interactions between HA and Ce6 following EDC/NHS coupling with HA. Infrared spectroscopy was utilized to verify the formation of covalent bonds between Ce6 and HA in HA-sese-Ce6 and to examine the binding interactions between HA and Ce6 following EDC/NHS coupling. The characteristic peaks for HA, such as O-H stretching, C-H stretching, amide I band, amide II band, C=O stretching and C-O stretching, were typically observed. The formation of amide bonds occurred through the EDC/NHS coupling process. The amide band in HA-sese-Ce6 displayed peaks at 3390, 1645, 1508 and 1101 cm^−1^, corresponding to N-H stretch combined with hydrogen bonding (amide A), C=O stretching (amide I), an N-H band coupled with a C=N stretching vibration (amide II), and an additional N-H band (amide III). While the peaks from HA and the peaks from amide bond overlapped in HA-sese-Ce6, they were distinct in Ce6-sese-HA, indicating successful incorporation of amide bonds and Ce6 into HA [23–25].

### Determination of crosslinker concentration

To determine the appropriate concentration of the PEGDE crosslinking agent required for the synthesis of the Ce6-HA hydrogel, we examined several factors, including the swelling ratio, enzyme stability and cytotoxicity. The quantity of the crosslinking agent had a significant impact on the physicochemical characteristics of the end product. However, excessive cross-linking agents may lead to toxicity; thus, determining the optimal concentration is crucial. PEGDE was chosen as the crosslinking agent due to its lower toxicity compared to other agents [26], and it was tested at three molar ratios: 0.3 mol PEGDE/1 mol HA, 0.5 mol PEGDE/1 mol HA and 0.7 mol PEGDE/1 mol.

**Figure 3.**
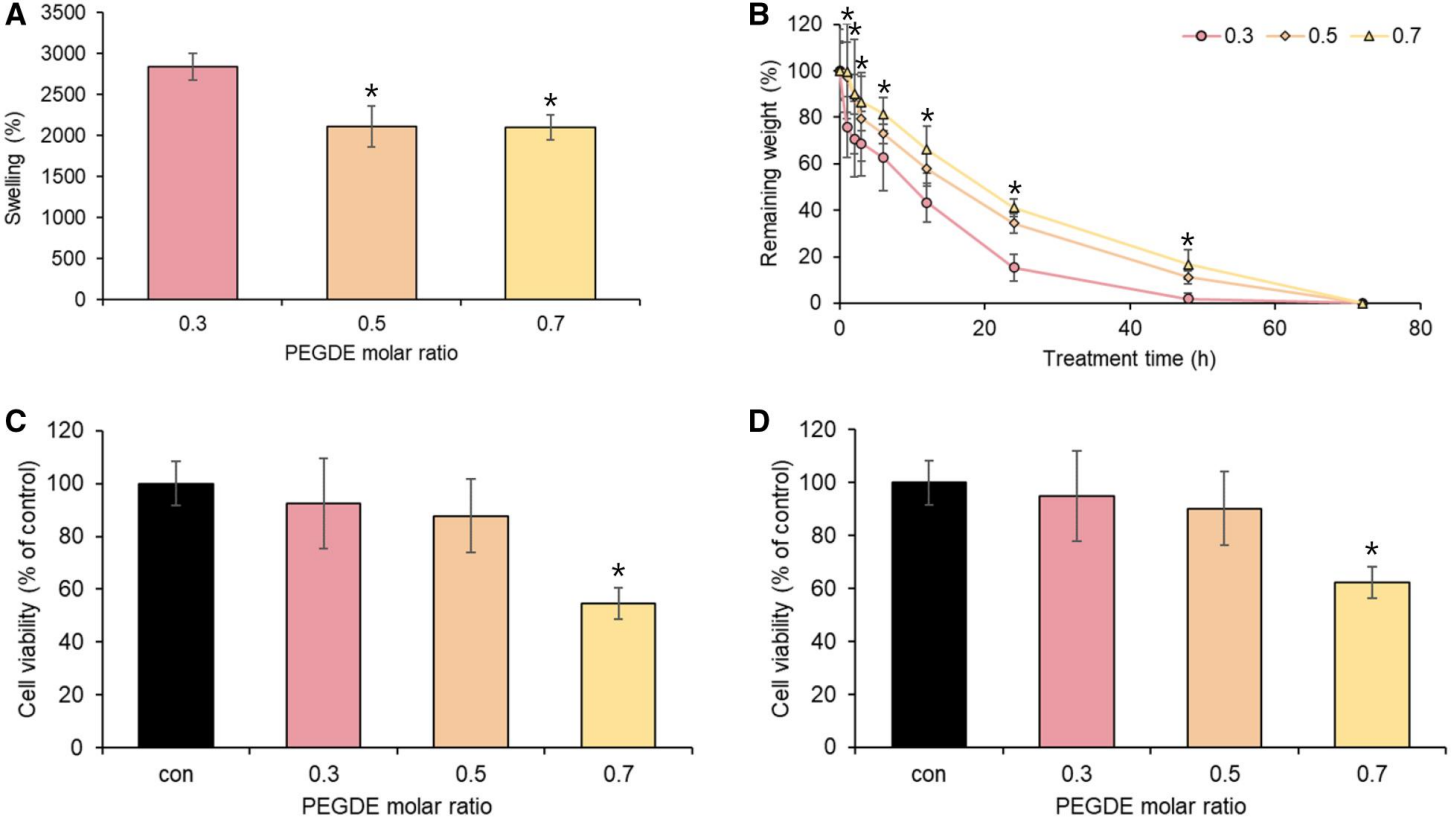
Crosslinking agent concentration determination. (**A**) Swelling was measured for Ce6-HA hydrogels formed with 0.3, 0.5 and 0.7 PEGDE molar ratios. The hydrogels were weighed, immersed in PBS for 48 h, and then weighed after immersion to calculate swelling ratios. Data were expressed as mean ± SD. *P < 0.05 vs. 0.3.(**B**) Enzymatic stability of Ce6-HA hydrogels formed with 0.3, 0.5 and 0.7 PEGDE molar ratios was determined by evaluating hydrogel degradation in a hyaluronidase solution over time. At each time point, Ce6-HA hydrogels were weighed and the weights at the timepoint were compared with the initial weights. Data were expressed as mean ± SD. *P < 0.05 vs. 0.3.(**C**) Cytotoxicity of Ce6-HA hydrogels formed with 0.3, 0.5 and 0.7 PEGDE molar ratios was determined by incubating the hydrogels with NHDF cells and then quantifying the number of viable cells by MTT assay. Data were expressed as mean ± SD. (**D**) Cytotoxicity of Ce6-HA hydrogels formed with 0.3, 0.5 and 0.7 PEGDE molar ratios was determined by incubating the hydrogels with HaCaT cells and then quantifying the number of viable cells by MTT assay. Data were expressed as mean ± SD. *P < 0.05 vs. con.

Swelling ratios are valuable indicators of hydrogel properties, as they allow us to indirectly assess the degree of crosslinking and observe how polymer side branches respond to environmental changes [27, 28]. To calculate the swelling ratios, we measured the weight of the hydrogels before and after immersion in PBS. The HA and Ce6-HA hydrogel, crosslinked with 0.3 mol PEGDE/1 mol HA, demonstrated the highest swelling ratio. When the concentration of crosslinker increased to 0.7 mol PEGDE/1 mol HA, the water absorption capacity of the HA and Ce6-HA hydrogel decreased. This can be attributed to the higher molar ratios of the crosslinker, which causes network expansion and requires connected chains to adopt a more elongated conformation, ultimately leading to reduced swelling capacity [29]. The swelling ratios showed that HA and Ce6-HA formed using 0.7 mol PEGDE/1 mol HA had a more solid structure than HA and Ce6-HA formed using 0.3 mol PEGDE/1 mol HA (Figure 3A).

The ability of a hydrogel to preserve its structural integrity and functional properties in the presence of enzymes is referred to as its enzyme stability [30]. Enzymes are biologically active substances that facilitate chemical reactions and can be found in biological environments, such as tissues and bodily fluids [31]. Enzyme stability is critical because it affects the effectiveness and longevity of hydrogels for various applications. Hyaluronidase, an enzyme known to break down HA, has been utilized in medical applications for over six decades, emphasizing the importance of confirming enzyme stability in HA gels [32]. In experiments involving different molar ratios of PEGDE cross-linked with HA-sese-Ce6 and treated with 100 units of hyaluronidase dissolved in PBS, the gels demonstrated varying degrees of degradation at different time points. Gels made with lower PEGDE ratios degraded more rapidly within 48 h, whereas those with higher PEGDE ratios remained stable until 72 h had elapsed. Additionally, gels made with a ratio of 0.5 mol PEGDE/1 mol HA showed similar enzyme stability to those made with a ratio of 0.7 mol PEGDE/1 mol HA (Figure 3B). These findings suggest that higher molar ratios of PEGDE may improve the stability of the hydrogel.

Cytotoxicity refers to the potential harm that hydrogel crosslinking agents may cause to living cells or tissues, which is an important consideration in biomedical applications as it establishes the biocompatibility and safety of the hydrogel [33, 34]. To evaluate the cytotoxicity of the material intended for use in contact with wounded skin, we employed NHDF and HaCaT skin cells along with PEGDE-crosslinked HA and Ce6-HA. To develop a HA hydrogel that could be applied to skin wounds, it was crucial to assess the toxicity of the crosslinker on skin cells. To achieve this, we used fibroblasts and keratinocytes, which are both representative skin cells, to evaluate the toxicity of the crosslinker. The gels created using PEGDE at molar ratios of 0.3 and 0.5, showed cellular viability exceeding 90%, indicating no significant variance in cytotoxicity. However, cell viability decreased when using a molar ratio of 0.7 for PEGDE (Figure 3C and D). Therefore, we selected a molar ratio of 0.5 mol PEGDE/1 mol HA for the crosslinking concentration based on a lower swelling ratio, longer endurance in enzymatic conditions and absence of cytotoxicity.

### ROS generation through LED irradiation

**Figure 4.**
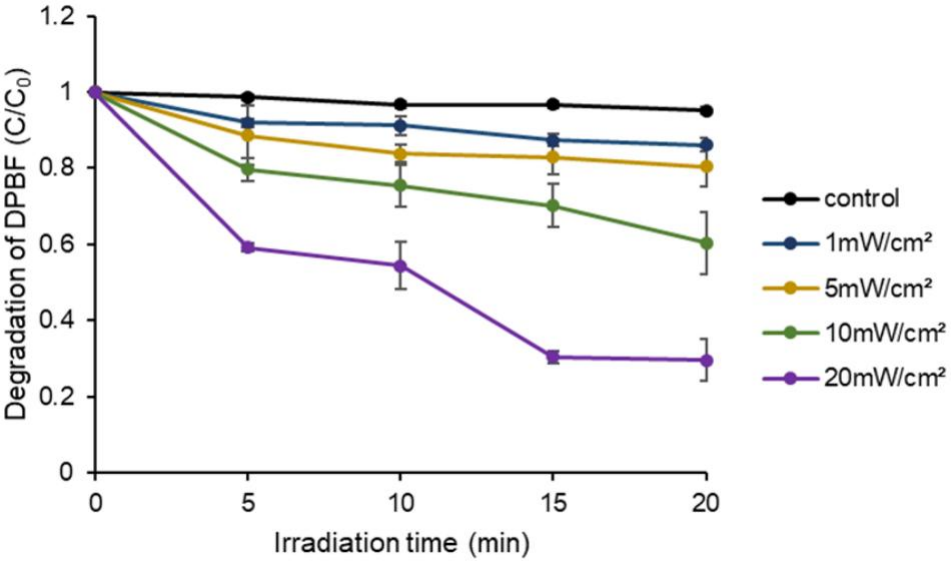
ROS generation of Ce6-HA through LED irradiation. ROS formation leads to the oxidation/degradation of DPBF. Oxidation/degradation of DPBF was measured upon LED irradiation at 1, 5, 10 and 20 mW/cm^2^ power every 5 min. Data were expressed as mean ± SD.

Ce6-HA induced ROS production under LED light exposure, as measured by the degradation of DPBF an indicator of ROS. The presence of ROS results in the transformation of yellow DPBF into a colorless compound [35]. The amount of ROS produced by the LED-irradiated Ce6-HA was determined by adjusting the LED power and irradiation time. Red LED irradiation was performed at different power levels every 5–10 min. The absorbance due to ROS generation was calculated as the *C*/*C*_0_ ratio, where *C_0_* represents the DPBF solution without light exposure. The degradation of DPBF was found to be directly proportional to both LED power and irradiation time (Figure 4).

### Verifying cytotoxicity and genetic mutation induced by ROS generated from Ce6-HA

**Figure 5.**
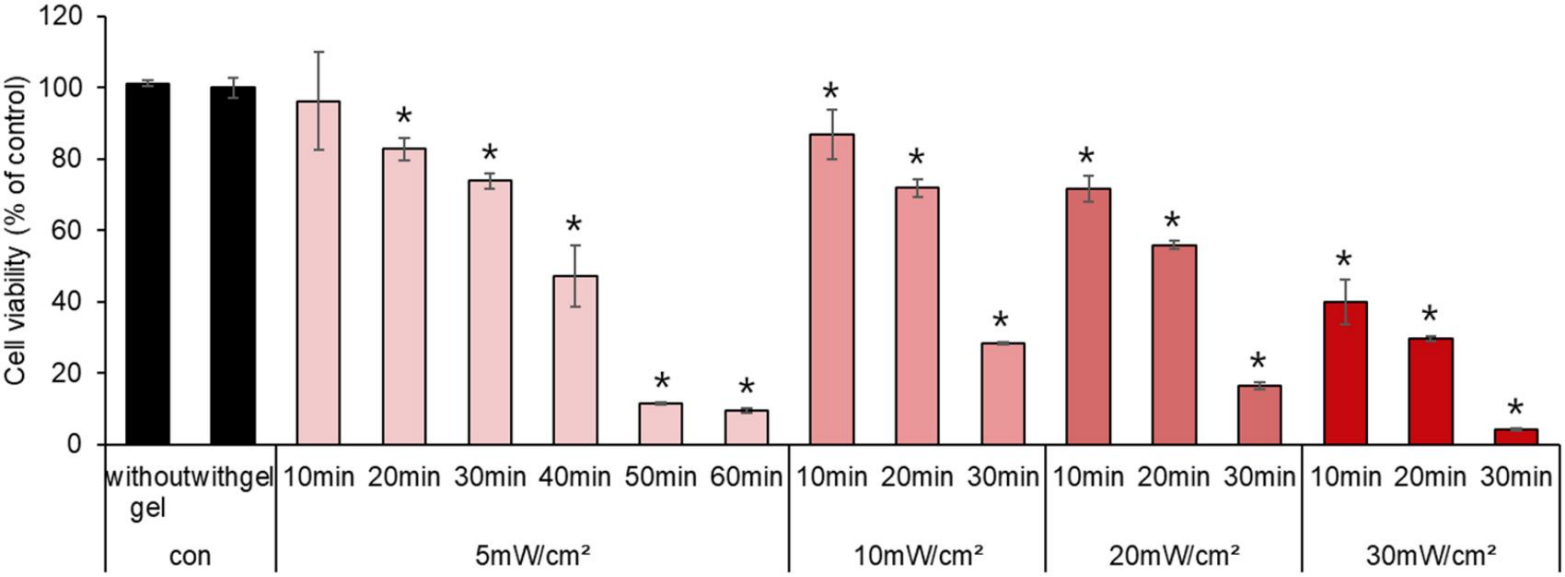
Biocompatibility of ROS generated from Ce6-HA. The cytotoxicity of the ROS generated from Ce6-HA irradiated with various LED intensities and times was evaluated by measuring viability of NHDF cells by MTT assay. The samples that were not irradiated were considered the controls. Data were expressed as mean ± SD. *P < 0.05 vs. control.

In our cytotoxicity study using NHDF skin cells, we aimed to identify conditions that would minimize damage to host tissue while maintaining cellular viability above 70% [36]. Specifically, we conducted LED irradiation at various power intensities and exposure times to evaluate the effects on cellular viability. At 30 mW/cm^2^ LED irradiation, a 10 min exposure reduced cellular viability to 39%, and longer exposure times led to further reductions. In contrast, at 20 mW/cm^2^ LED irradiation, a 10 min exposure resulted in 71% cellular viability, and 20–30 min exposure reduced viability to under 60%. At 10 mW/cm^2^ LED irradiation, 10–20 min exposure maintained cellular viability above 70%, but 30 min exposure reduced viability to 34%. At 5 mW/cm^2^ LED irradiation, cellular viability remained above 70% for 10–30 min of exposure, but decreased to under 50% for 40–60 min of exposure (Figure 5). Based on these findings, we recommend 5 mW/cm^2^ for 10–30 min and 10 mW/cm^2^ for 10–20 min as the most maximum LED exposure parameters to minimize cell damage.

Researchers typically use the Ames test to assess the potential of different substances to cause genetic mutations. This test involves inducing reverse mutations in specific strains of *Salmonella typhimurium* and *E.coli* with respect to certain amino acids [37]. In this study, we investigated various factors that could potentially cause mutations, including LED light exposure, HA gel, and ROS. The experiment included five test groups: negative control, positive control, HA irradiation with LED light, Ce6-HA non-irradiated and Ce6-HA irradiation with LED light. The samples were then exposed to a 10 mW/cm^2^ LED for 20 min. There were significant variations in the revertants between the negative and positive control groups. When comparing the HA and Ce6-HA groups with or without irradiation to the positive control group, notable differences were observed; however, when compared to the negative control, no significant differences were found (Table 1). Consequently, it was concluded that neither LED light nor compounds from the hydrogel induced mutations, indicating a promising safety profile that supports the potential use of LED-exposed compounds.

**Table 1.** Confirmation of genotoxicity through ROS

	TA98	TA100	TA1535	TA1537	*Escherichia coli*
HA negative	26 ± 2.8	65.3 ± 3.2	8.3 ± 1.5	9 ± 3.5	111 ± 22.5
HA positive	93.7 ± 9.0	810.7 ± 91.0	821.7 ± 68.2	1390.7 ± 164.1	2066.0 ± 258.2
HA + LED	20.5 ± 3.5	55.0 ± 7.5	14.7 ± 4.5	10 ± 4.4	120.7 ± 20.6
Ce6-HA	12.7 ± 5.9	65.3 ± 8.6	13.0 ± 2.6	7.3 ± 2.5	95.7 ± 11.5
Ce6-HA + LED	20.7 ± 6.5	72 ± 4.4	10.3 ± 3.8	10.7 ± 6.5	103.0 ± 6.0

### ROS effect for antibacterial effect

**Figure 6.**
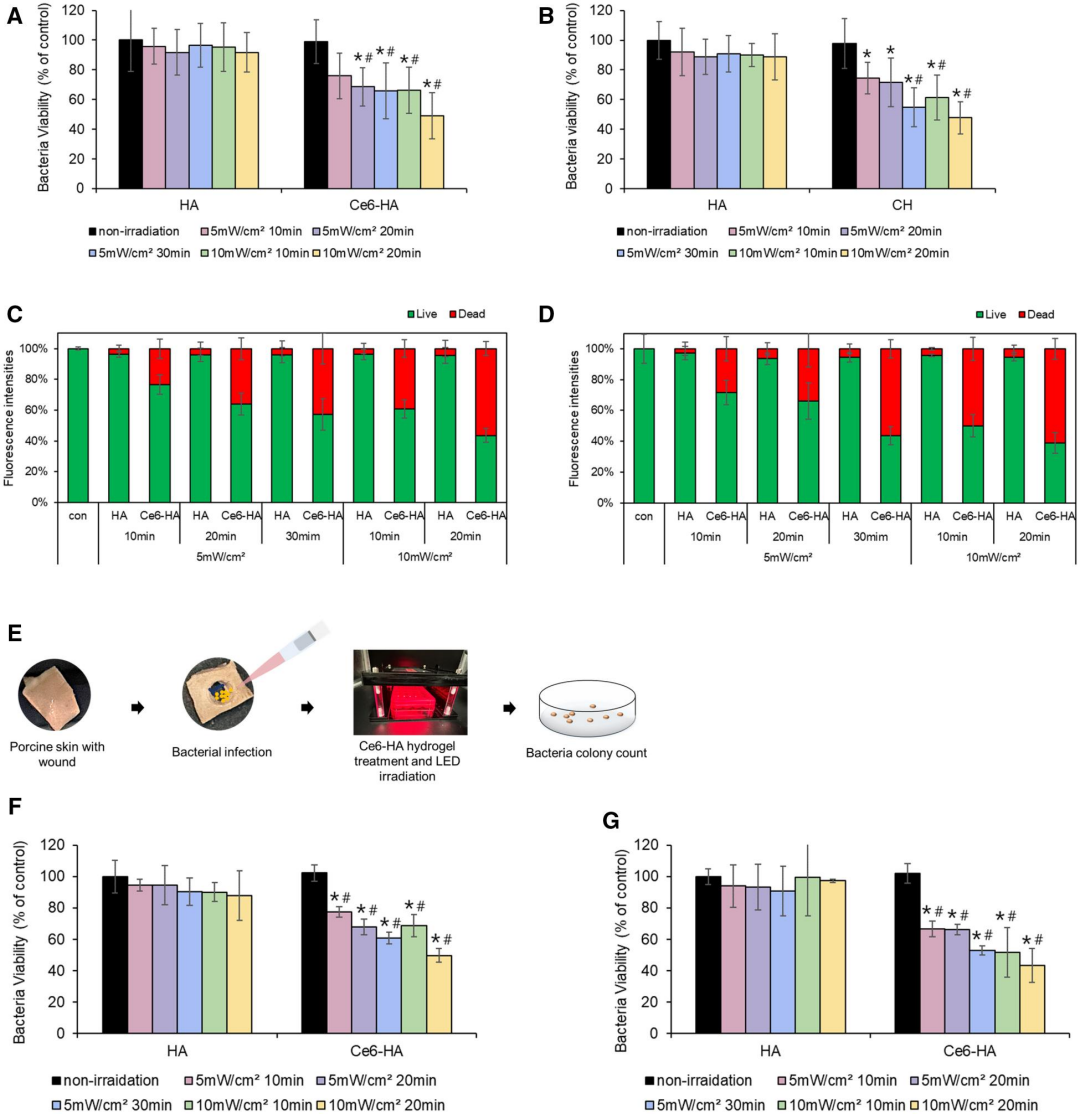
Antibacterial effect of Ce6-HA hydrogel at *in vitro* and *ex vivo* model. (**A**) *S.aureus* and (**B**) *P.aeruginosa* were treated with HA and Ce6-HA under various LED irradiation conditions. After irradiation, cell suspensions were spread on agar plates and colonies were counted manually. The number of counted colonies was expressed as a percent of the control. Data were expressed as mean ± SD. *P < 0.05 vs. control, ^#^*P* < 0.05 vs. HA. (**C**) *S.aureus* and (**D**) *P.aeruginosa* fluorescence intensity was presented as percent of control. (**E**) Schematic of the *ex vivo* experiment about bacterial infected porcine skin. (**F**) *S.aureus* and (**G**) *P.aeruginosa* was infected with each bacterial at porcine skin. HA or Ce6-HA was treated and LED was irradiated. The results were expressed as a percentage in comparison to the control group. *P < 0.05 vs. control, ^#^*P* < 0.05 vs. HA.

Two types of microorganisms were utilized: *P.aeruginosa* and *S.aureus*. These two bacterial strains are the most prevalent causes of chronic wound infections and are frequently found in conjunction, representing Gram-negative and Gram-positive organisms, respectively. Bacterial suspensions at a concentration of 5 × 10^6^ CFU/ml were treated with either HA or Ce6-HA followed by LED irradiation. LED irradiation was carried out at intensities of 5 and 10 mW/cm^2^, at intervals of 10 min, based on the cellular cytotoxicity test. The control group did not undergo LED irradiation. Upon comparison between the LED-irradiated HA and control groups, no significant differences were observed under all conditions, suggesting the absence of antibacterial effects. However, a notable decrease in bacterial survival was observed in the group exposed to LED light with Ce6-HA compared with that in the control group. After treatment with Ce6-HA and subjected to a 20 min exposure to LED at 10 mW/cm^2^, both *S.aureus* and *P.aeruginosa* exhibited viabilities lower than 50% (Figure 6A and B). To substantiate the bacterial elimination effect caused by ROS, fluorescence staining was conducted to determine the proportion of live and dead bacteria. Quantitative analysis confirmed results that were consistent with those obtained from the plate count assay (Figure 6C and D). ROS produced by LED-irradiated Ce6-HA contributed to reduced bacterial viability. Therefore, it can be concluded that combining Ce6-HA hydrogel with LED light shows promise as a strategic antibacterial approach.

The implementation of an *ex vivo* model of infection with bacteria using porcine skin was carried out based on the findings of an *in vitro* experiment. This *ex vivo* investigation aimed to validate these outcomes in a real clinical environment (Figure 6E). As the intensity and duration of light exposure increased, bacterial mortality rate also increased. Specifically, when exposed to 10 mW/cm^2^ for 20 min, more than half of the microorganisms were eradicated, which aligns with the *in vitro* findings (Figure 6F and G). This consistency in results across different substrates highlights the significance of applying ROS to eliminate microbes at the onset of a wound infection, demonstrating its potential efficacy.

**Figure 7.**
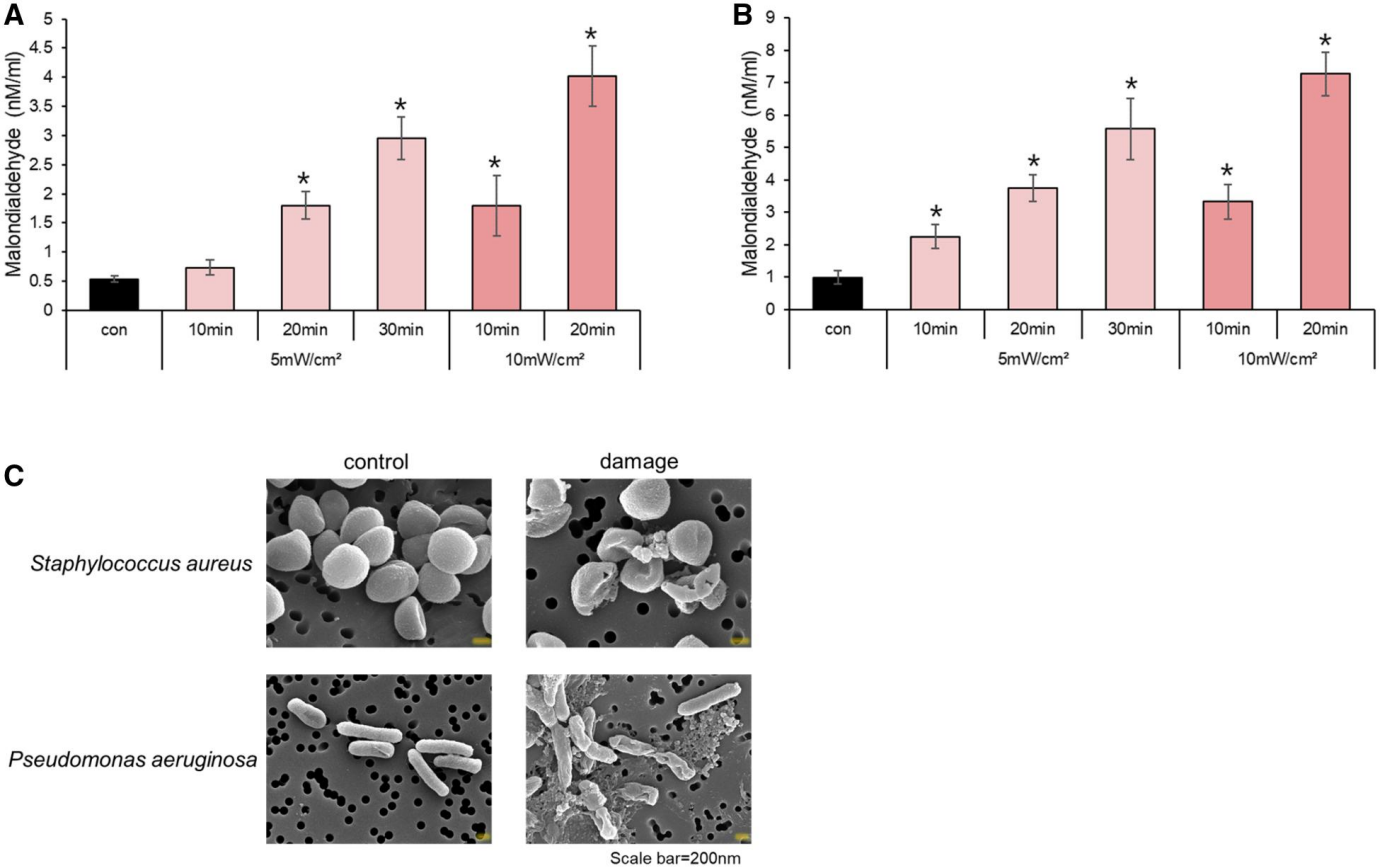
Antibacterial effect of ROS membrane damage effect. (**A**) *S.aureus* and (**B**) *P.aeruginosa* were treated with HA and Ce6-HA and then LED irradiated. Then, the cells were tested for their MDA levels as described in the Materials and methods. Data were expressed as mean ± SD. *P < 0.05 vs. control. (**C**) SEM images of *S.aureus* and *P.aeruginosa* in the absence of ROS (control) and in the presence of ROS (damaged). Scale bar = 200 nm.

One consequence of ROS is lipid peroxidation, which results in the generation of MDA [38–40]. Untreated *S.aureus* contained 0.5 nM/ml MDA. However, there was a notable increase in the MDA concentration in *S.aureus* cells after exposure to LED-irradiated Ce6-HA (Figure 7A). Untreated *P.aeruginosa* showed 1 nM/ml MDA, but the MDA concentration significantly increased in *P.aeruginosa* following exposure to LED-irradiated Ce6-HA (Figure 7B). These findings demonstrated that the amplified production of ROS through LED-irradiated Ce6-HA led to increased lipid peroxidation in both *S.aureus* and *P.aeruginosa*, resulting in elevated levels of MDA.

The surfaces of *S.aureus* and *P.aeruginosa* were analyzed before and after exposure to ROS. The untreated bacteria exhibited smooth circular surfaces with distinct boundaries. However, upon exposure to ROS, visible vesicles appeared on the surfaces and the integrity of the surface membranes was compromised. These modifications suggest cellular damage caused by ROS-induced structural changes in bacteria, which may ultimately result in bacterial death [41, 42]. The changes in morphology observed in the ROS-treated bacteria support the antibacterial properties of the LED-irradiated Ce6-HA hydrogel (Figure 7C).

Colony counting, the MDA assay and SEM images revealed that Ce6-HA, when exposed to LED irradiation, generated ROS, which resulted in bacterial death and membrane destruction. The antibacterial mechanism of action of ROS involves oxidative stress that damages membranes, proteins, nucleic acids and enzymes, leading to disruption of the integrity and function of the microbe and ultimately causing death.

Previously, various materials with antibacterial properties have been utilized, including silver nanoparticles, metal-organic frameworks, antibiotics and natural antibacterial agents [43–46]. However, these materials have limitations, such as potential cytotoxicity, safety concerns and antibiotic resistance. The platforms we utilize generate high levels of ROS in response to light and are effective against both Gram-negative and Gram-positive bacteria. The localized action of these platforms allows ROS production to be activated by light, minimizing damage to the surrounding tissues. Additionally, Ce6 can be activated by specific wavelengths of light, allowing precise control over the timing and location of ROS production.

### ROS-induced macrophage polarization

**Figure 8.**
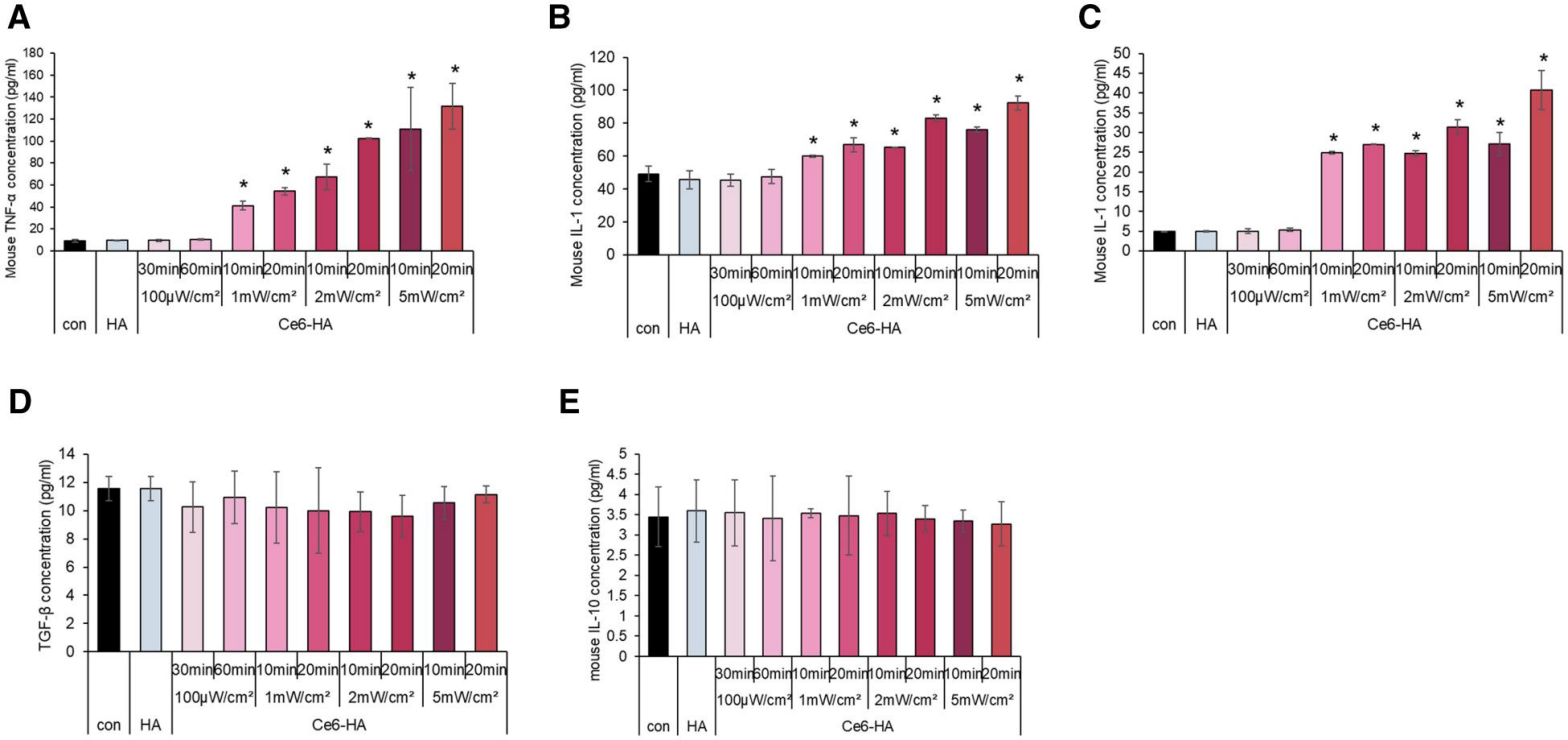
Induction of pro- and anti-inflammatory cytokine secretion by ROS. (**A**) Pro-inflammatory mouse TNF-α, (**B**) pro-inflammatory mouse IL-1β, (**C**) pro-inflammatory mouse IL-6, (**D**) anti-inflammatory mouse TGF-β and (**E**) anti-inflammatory mouse IL-10 levels were measured by ELISA. Data were expressed as mean ± SD. *P < 0.05 vs. HA.

The RAW 264.7 line were grown in a well plate and exposed to HA or Ce6-HA treatment. LED light with varying intensities of 1, 2 and 5 mW/cm^2^ was applied for periods ranging from 10 to 20 min. The results showed that the group that received Ce6-HA treatment had significantly higher levels of pro-inflammatory cytokines, including TNF-α, IL-1β and IL-6, than the HA-treated group. This suggests a shift in macrophage polarization towards the M1 phenotype. However, both groups exhibited no variation in the levels of the anti-inflammatory cytokines, TGF-β and IL-10. When stimulated with 100 μW/cm^2^ for 30–60 min, there was no difference between the HA-treated and Ce6-HA-treated groups in pro-inflammatory and anti-inflammatory cytokines (as shown in Figure 8A–E). These data indicate that the generation of ROS stimulates the production of pro-inflammatory cytokines by macrophages, serving as a defensive response to invading pathogens [47–49]. However, a minimal level of ROS does not alter macrophage activity [50]. This demonstrates that the response of macrophages to ROS stimulation varies depending on the level of ROS. It is evident that a specific quantity or higher level of ROS influences macrophage polarization. Even a minimal quantity of ROS that is less than the effective level against bacteria can affect macrophage polarization. It is plausible that macrophage polarization plays a role in the host defense mechanism under antibacterial conditions [51]. These findings contribute to our understanding of the Ce6-HA hydrogel and its potential application in boosting the immune response of the body to pathogens. ROS play a role in the polarization of macrophages, which are multifunctional immune cells that exhibit unique characteristics in response to various stimuli and environments [52]. Macrophages can exist in two primary polarization states: pro-inflammatory M1 phenotype and anti-inflammatory M2 phenotype. Initially, during wound healing and the body’s inflammatory response, immune system cells generate endogenous ROS by stimulating enzymes, such as NADPH oxidase. These ROS contribute to guiding macrophages towards the M1 phenotype, which is associated with an inflammatory reaction. It has been suggested that M1 macrophages, which play a role in the inflammatory response, transform into M2 macrophages when the inflammation resolves, thereby supporting the formation of new blood vessels. Polarizing towards M1, which demonstrates a robust ROS reaction in response to pathogen invasion, could expedite the resolution of inflammation [53]. Macrophages polarized to M1 are highly phagocytic, engulfing and digesting bacteria and other pathogens, and produce ROS, which are toxic to bacteria. In addition, M1 macrophages secrete antimicrobial peptides that can directly kill bacteria, and the inflammatory cytokines they produce recruit and activate other immune cells to the site of infection, enhancing the overall immunity against pathogens. Therefore, the polarization of macrophages to M1 results in more rapid bacterial killing [54, 55].

**Figure 9.**
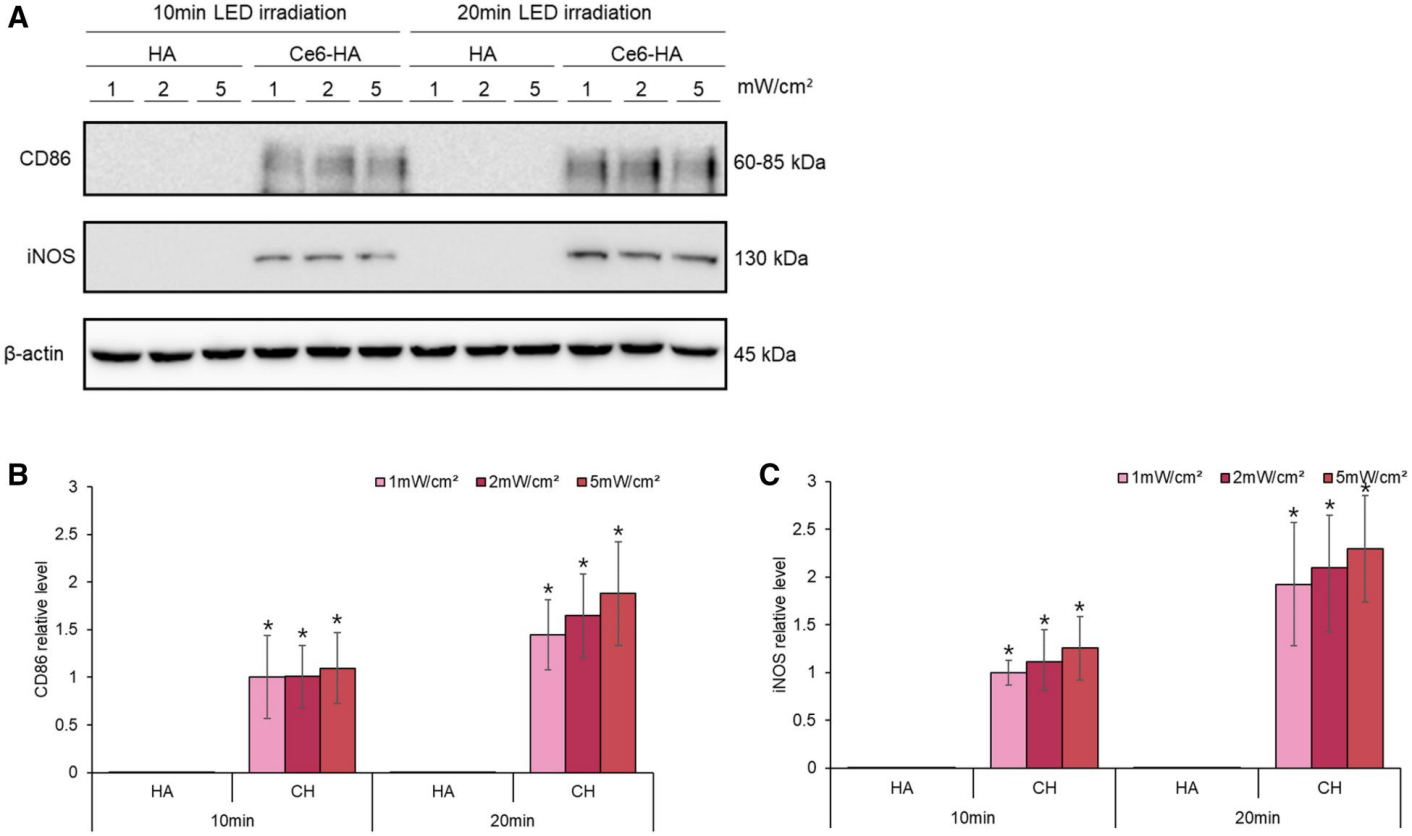
The expression of iNOS and CD86 in macrophages treated with hydrogel and LED. (**A**) RAW 264.7 with HA or Ce6-HA was irradiated with LED. Samples were collected and protein expression of CD86, iNOS was detected. The graphs showed the western blot quantification of (**B**) CD86 and (**C**) iNOS. The data are expressed as means ± SD. *P < 0.05 vs. HA.

Given the importance of iNOS and CD86 regulation at the transcriptional level, we conducted western blot analysis to examine their protein levels [56]. Our results revealed that cells treated with Ce6-HA showed higher levels of iNOS and CD86 protein than those treated with HA (Figure 9A–C). This finding suggests that increased levels of ROS may affect macrophage polarization towards the M1 phenotype through cytokine release and protein expression. Elevated ROS levels can activate transcription factors, such as NF-κB and signal transducer and activator of transcription 3, leading to the production of inflammatory cytokines, chemokines and inducible nitric oxide synthase (iNOS). Therefore, it is critical to regulate ROS levels and their interactions with signaling pathways to maintain appropriate immune responses [57, 58]. ROS can activate various signaling pathways, including the NF-κB, MAPK and JAK-STAT pathways, to increase the expression of inflammatory genes [59–62]. ROS activates IκB kinase to degrade IκB proteins, and NF-κB translocates to the nucleus to promote the transcription of inflammatory genes. ROS also activates MAPK signaling pathways, including p38 MAPK and JNK, which promote the expression of proinflammatory cytokines and other genes. ROS increases the expression of proinflammatory genes through the JAK-STAT pathway and enhances M1 polarization. ROS also promotes macrophage polarization, and polarized M1 macrophages produce more ROS to maintain their polarized state, which has potent antimicrobial effects.

## Conclusion

Ce6 was successfully bonded to HA and its properties were confirmed, with no observed negative effects on cells. Chemical linkage between Ce6 and HA was achieved through the bonding of the functional groups present on both molecules. Various methods for characterizing materials, such as spectroscopic analysis, have been used to verify the formation and structural integrity of hybrid substances. Subsequent evaluations of cell viability and genotoxicity using assays indicated that the linked Ce6-HA complex was safe for use in biomedical settings.

Analysis of suitable LED light wavelengths resulted in the generation of ROS, which exhibited direct antibacterial effects. ROS eliminate bacterial pathogens and stimulate macrophages to secrete cytokines that enhance antibacterial activity and facilitate the removal of microbes. This study demonstrated that ROS play a dual role in protecting against microbes: they directly eliminate pathogens and stimulate immune responses. This comprehensive approach highlights the potential of Ce6-conjugated HA in PDT, providing a promising strategy for targeting and treating bacterial infections through the combined use of direct antibacterial effects and immune system activation.
